# Supplementary material for: Association between a complex community intervention and quality of health extension workers’ performance to correctly classify common childhood illnesses in four regions of Ethiopia
Source: PLoS One. 2021 Mar 12;16(3):e0247474. doi: 10.1371/journal.pone.0247474 (PMC7954333; doi:10.1371/journal.pone.0247474)
Supplement: S1 Table — (DOCX) [file pone.0247474.s002.docx]

S1 table: Theory of change for OHEP interventions
