# Supplementary material for: Association between a complex community intervention and quality of health extension workers’ performance to correctly classify common childhood illnesses in four regions of Ethiopia
Source: PLoS One. 2021 Mar 12;16(3):e0247474. doi: 10.1371/journal.pone.0247474 (PMC7954333; doi:10.1371/journal.pone.0247474)
Supplement: S1 Protocol — A before and after study in intervention and comparison areas. (DOCX) [file pone.0247474.s003.docx]

Protocol for the evaluation of a complex intervention aiming at increased utilisation of primary child health services in Ethiopia. A before and after study in intervention and comparison areas

Contents

[Summary 3](#_Toc34387572)

[Background 5](#_Toc34387573)

[Design and methods 7](#_Toc34387574)

[Setting 7](#_Toc34387575)

[Barrier analysis 8](#_Toc34387576)

[The intervention and underlying assumptions 11](#_Toc34387577)

[Implementation 11](#_Toc34387578)

[Process evaluation, including mechanisms of impact, and contextual factors 12](#_Toc34387579)

[Effectiveness of the Optimizing the Health Extension Program 14](#_Toc34387580)

[Outcomes 16](#_Toc34387581)

[Ethics approval and consent to participate 20](#_Toc34387582)

[Conclusion 21](#_Toc34387583)

## Summary

**Background:** By expanding primary health care services, Ethiopia has reduced under-five mortality. Utilisation of these services is still low, and concerted efforts are needed for continued improvements in newborn and child survival. “Optimizing the Health Extension Program” is a complex intervention based on a logic framework developed from an analysis of barriers to the utilisation of primary child health services. This intervention includes innovative components to engage the community, strengthen the capacity of primary health care workers, and reinforce the local ownership and accountability of the primary child health services. This paper presents a protocol for the process and outcome evaluation, using a pragmatic trial design including before-and-after assessments in both intervention and comparison areas across four Ethiopian regions. The study has an integrated research capacity building initiative, including ten Ph.D. students recruited from Ethiopian Regional Health Bureaux and universities.

**Methods:** Baseline and endline surveys two years apart include household, facility, health worker, and district health office modules in intervention and comparison areas across Amhara, Southern Nations Nationalities and Peoples (SNNP), Oromia, and Tigray regions. The effectiveness of the intervention on the seeking and receiving of appropriate care will be estimated by difference-in-differences analysis, adjusting for clustering and for relevant confounders. The process evaluation follows the guidelines of the UK Medical Research Council. The implementation is monitored using data that we anticipate will be used to describe the fidelity, reach, dose, contextual factors and cost. The participating Ph.D. students plan to perform in-depth analyses on different topics including equity, referral, newborn care practices, quality-of-care, geographic differences, and other process evaluation components.

**Conclusion:** This protocol describes an evaluation of a complex intervention that aims at increased utilisation of primary and child health services. This unique collaborative effort includes key stakeholders from the Ethiopian health system, the implementing non-governmental organisations and universities, and combines state-of-the art effectiveness estimates and process evaluation with capacity building. The lessons learned from the project will inform efforts to engage communities and increase utilisation of care for children in other parts of Ethiopia and beyond.

Trial registration: Current Controlled Trials ISRCTN12040912, retrospectively registered on 19 December, 2017. http://www.isrctn.com/ISRCTN12040912

## Background

Ethiopia reached the Millennium Development Goal 4, reflected in a reduction of the under-five mortality from 205 deaths per 1000 live births in 1990 to 64 in 2014 (1). Neonatal mortality also decreased from 55 to 28 deaths per 1000 live births in the same period. The expansion of primary care services, improvements in nutrition (2), and progress across other sectors of society have reportedly contributed to reaching this goal. An analysis based on the Global Burden of Disease study reported neonatal conditions, together with lower respiratory tract infection and diarrhoeal diseases, as the dominant causes of under-five death (3).

As part of the Health Extension Program, in 2003 the Ethiopian Government introduced a new cadre of primary care workers called Health Extension Workers (HEWs)to reinforce efforts to improve maternal, newborn and child health in Ethiopia (4). This category of health workers is reportedly able to correctly manage multiple child illnesses through the integrated community case management (iCCM) activities that the government initiated in 2010 (5). If appropriately trained and supported, this cadre can also treat severe infections of the newborn in a cost-effective way within the Community-Based Newborn Care (CBNC) program that has been running since 2014 (6). Although nearly all the HEWs throughout the country have been trained, relatively few sick newborns have been identified and treated(7). Since 2011 a volunteer cadre called the Women’s Development Army (WDA-also known as the Health Development Army or Women’s Development Group) is active in promoting the use of health services (8,9). There are two levels to the WDA leaders. The smaller unit is comprised of 6 women, with one serving as a leader. Five or six of these networks are combined to form a group. The approximately 30 women in one group are led by one of the network leaders. The WDA leaders work closely with HEWs to promote maternal and child health services.

In Ethiopia, the utilisation of maternal health services shows disparities between regions and social groups (10,11), and skilled birth attendance, although increasing, has remained inadequate with increasing social inequity (12). Insufficient number of sick children are taken to HEWs for treatment of common childhood diseases, and the expansion of the iCCM program has not created sufficient community demand for use of these services (13).

The low utilisation of child health care calls for concerted efforts to improve the equitable reach of high-quality services (14,15). This background formed the rationale for developing a complex intervention to engage the community, strengthen the capacity of primary care workers to provide high-quality services and reinforce the local ownership and accountability of the primary level maternal and child health services. It was hypothesized that, given the low service utilization, increased awareness and promotion of primary health services in combination with strengthened provision of iCCM and CBNC and improved ownership and accountability of these services would lead to increased service use, which ultimately would further reduce neonatal and under-five mortality.

Thus, the evaluation described in this protocol aims to assess whether the proportion of children under the age of five years with suspected pneumonia, diarrhoea, fever or neonatal sepsis, that seek and receive appropriate care has increased in intervention as compared to comparison areas. Further, we present a plan for whether the information on the fidelity, reach, and dose of the interventions support the plausibility of changes in the outcome, and provide insights into the pathways of effects, feasibility, and program cost-effectiveness. Findings of this evaluation will be used to improve existing platforms of service delivery in the country. It is anticipated that successful interventions will be scaled up nationally provided sufficient budget support is available.

## Design and methods

### Setting

The Federal Ministry of Health in collaboration with the non-governmental partners UNICEF, Last 10 Kilometres/John Snow, Inc., Save the Children, and PATH initiated the “Optimizing the Health Extension Program” project to increase the utilisation of primary child health services. This evaluation has a pragmatic trial design with purposefully selected 26 intervention and 26 comparison districts (woredas), with a total population of 8 million, across four regions of Ethiopia (Amhara, Southern Nations Nationalities and People, Oromia, and Tigray). Figure 1 shows the intervention and comparison areas within these four regions.


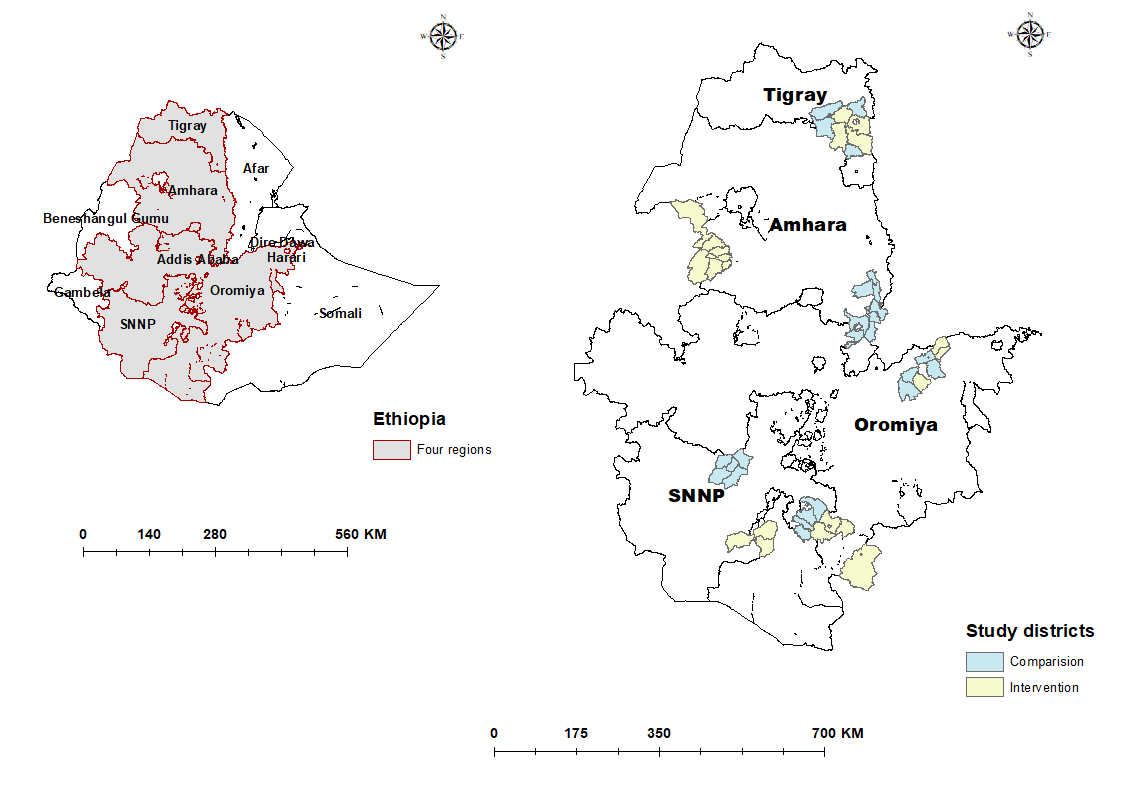


Figure 1. Map of Ethiopia showing all regions (left) and the intervention and comparison districts within the four study regions (right).

The intervention areas are selected by government and implementing partners for having a relatively low utilization of primary child health services. The Regional Health Bureaus in these regions, with the support from their local universities, will select the comparison districts to match the intervention districts. Selection is based on demographic and health criteria that includ population size, number of primary health care units, burden of diseases, health service performance data, length of time since iCCM and CBNC program initiation, prior exposure to other similar programs, and absence of non-governmental organizations addressing demand generation. The intervention, which will start in 2016, has an intended duration of 2.5 years and is based on an analysis of barriers to the utilisation of newborn, child and maternal health services. The planned evaluation follows a plausibility approach (16). It includes analysis of difference in differences of outcomes and a process evaluation of the intervention in line with the UK Medical Research Council’s guidelines (17).

The baseline and endline surveys, as well as the process evaluation are implemented by the London School of Hygiene & Tropical Medicine (LSHTM) and Ethiopian Public Health Institute (EPHI) along with representatives from Gondar, Jimma, Mekelle and Hawassa Universities. A steering committee comprising representatives from each of the universities, implementing partners, Ethiopian Public Health Institute and Federal Ministry of Health will be established to meet quarterly. The committee will provides advice on the evaluation of the project and assists in resolving issues encountered during the course of the evaluation. Given that the Optimising the Health Extension Program is a community and health system level intervention, a data monitoring committee is not deemed necessary.

### Barrier analysis

Key Ethiopian governmental and non-governmental stakeholders in the field of maternal, newborn and child health services met in 2016 for a facilitated workshop where the perceived demand- and supply-side barriers to CBNC and iCCM service utilisation were identified. The demand-side barriers included perceived lack of knowledge of diseases and danger signs (18), and lack of awareness of what primary level services could offer. Further, it was suggested that families often have a preference for traditional healers and home remedies (19) and that the higher availability of services offered by private providers was also appreciated in the households (20). In a qualitative analysis of barriers to care-seeking for common childhood infectious diseases, the trust in the primary care services was low (21). The barrier analysis showed a lack of community awareness of the curative, as well as preventive services provided by the HEW and that the quality of care on the primary level was perceived to be low (22). Also, there was a felt need to strengthen the HEWs in supporting pregnant women in birth preparedness, referral to midwives, and institutional delivery (23). All the above listed barriers resulted in under-use of maternal, newborn and child health services. Other demand side barriers included delay in seeking care due to the need to obtain husbands’ permission and financial support, the perceived cost and real cost of travel, particularly due to costs associated with referral of severely ill children to a further facility and repeated travel due to health post closure (21). Supply-side barriers included frequent stock-outs of medicines and other necessary supplies, service interruption and inconsistent operating hours at health posts (24). Further, it showed that HEWs had poor skills and confidence (25), especially when managing and treating newborns (26), lack of local government ownership and lack of accountability for both the CBNC and iCCM programs, and inconsistent supervision and monitoring. This barrier analysis formed the basis of a logic framework for a complex intervention that postulated that community engagement would increase care seeking for ill children, capacity building would improve availability of quality of CBNC and iCCM services and district level ownership and accountability would improve integration of these services into the district level planning and budgeting (Table 1). Together these three strategies would lead to an increased utilization of CBNC and iCCM services.

Table 1. Logic framework for the Optimizing Health Extension Program intervention in selected districts of Ethiopia.

| Assumptions | - Local stakeholders committed to coordinate and support the interventions - Traditional leaders will promote the maternal, newborn and child health services - The government health sector and supply chain partners will ensure drug and service availability | | |
| --- | --- | --- | --- |
| Strategies | COMMUNITY ENGAGEMENT | CAPACITY BUILDING | OWNERSHIP, ACCOUNTABILITY |
| Interventions | - Health post open house - Group discussions led by Women’s Development Army (WDA) members - Reaching male partners - Engaging schools - Engaging religious and traditional leaders - Health films - Radio spots and dramas | - WDA level one training - Community-based data for decision making - Health Extension Worker (HEW) gap filling training and job aids - Supportive supervision of HEWs - Performance review and mentorship meetings with HEWs - Provision of job aids and tools | - Advocacy for the integration of Community-Based Newborn Care (CBNC) and integrated community case management (iCCM) into planning, budgeting, management, and information systems of the district and sub-district levels. - Management standard for health post opening hours - Ambulance service for children’s referral - Engage Kebele (sub-district) command post in the efforts   Establish community feedback mechanism |
| Output | - Awareness of childhood illness and availability of CBNC and iCCM - Acceptance of health post care - Evidence-based social and behavioural change communication | - WDA members capacitated - HEWs gained skills - Supportive supervision and performance review and mentorship meetings with HEWs done | - CBNC and iCCM integrated in the planning, management and information systems at district and sub-district levels - Standard set for health post opening hours - Sub-district level local administration engaged in demand creation and support to primary health service provision - Community feedback mechanisms created - Advocacy to decision makers and influential bodies |
| Intermediate outcomes | - Improved child health practice at household and community levels   Data source: Household module   - Improved availability of high quality community-based newborn care and integrated community case management of childhood diseases   Data source: Health post, health extension worker and health provider assessment module   - Improved ownership and accountability of community-based newborn care and integrated management of childhood illnesses   Data source: woreda contextual factors module | | |
| Outcome | - Increased utilisation of good quality community-based newborn care and integrated management of childhood illnesses   Data source: household module | | |
| Impact | - Reduction of under-five mortality | | |

### The intervention and underlying assumptions

The package of interventions to be implemented across 26 districts includes three interlinked strategies with possible synergies: (1) community engagement activities that aim at increasing the awareness of newborn and child diseases, the recognition and acceptance of the care provided on the primary level, and the formulation of action plans at the local level; (2) capacity building of HEWs and WDA leaders such as gap filling training, supportive supervision and mentorship to improve iCCM and CBNC services, and (3) strengthening the local government’s ownership and accountability of the primary newborn and child health services by advocating for the sustained integration of CBNC and iCCM into the planning, budgeting, monitoring, management and support systems of the district and sub-district level (Table 1). Assumptions made by the implementers to achieve the success of the Optimizing the Health Extension Program included support from local stakeholders, traditional and religious leaders, governmental health sector and supply chain partners.

### Implementation

The Ethiopian Government, in collaboration with PATH and UNICEF (through sub-contractors Save the Children and Last 10 Kilometres) will implement the intervention. These organizations will have quarterly meetings to harmonize the intervention activities across the 26 intervention districts.

Trained professionals from the implementing organizations and the public sector will facilitate the community engagement activities (Table 1). To raise community awareness of iCCM and CBNC services, implementers will organise health post open house sessions to introduce available services and conduct workshops with schoolteachers and religious leaders. Community engagement will also be supported by behaviour change communication materials (brochures, posters, and banners). Educational films developed by implementers will be screened in health facilities and similarly radio messages and dramas will be developed and broadcast in implementation districts. Health professionals within the district health services facilitate the capacity building activities. For example, midwives from health centres provide training to HEWs, while HEWs provide training for WDA leaders. Where missing, implementers will provide registration books, iCCM and CBNC treatment algorithm booklets and backpacks to carry these items for community level service provision. Facilitators from the implementing partners lead the ownership and accountability activities. District ownership efforts include advocacy workshops at district and sub-district levels and support to the annual district health planning sessions.

The implementers plan to achieve a high reach of the different innovations across all intervention districts. Thus, the ambition is that the community meetings, school engagement activities, training of WDA leaders and HEWs, as well as district ownership efforts should reach all areas and relevant stakeholders.

### Process evaluation, including mechanisms of impact, and contextual factors

The process evaluation is guided by the UK Medical Research Council framework for complex interventions (17). A graphical representation of the process evaluation is provided in Figure 2.


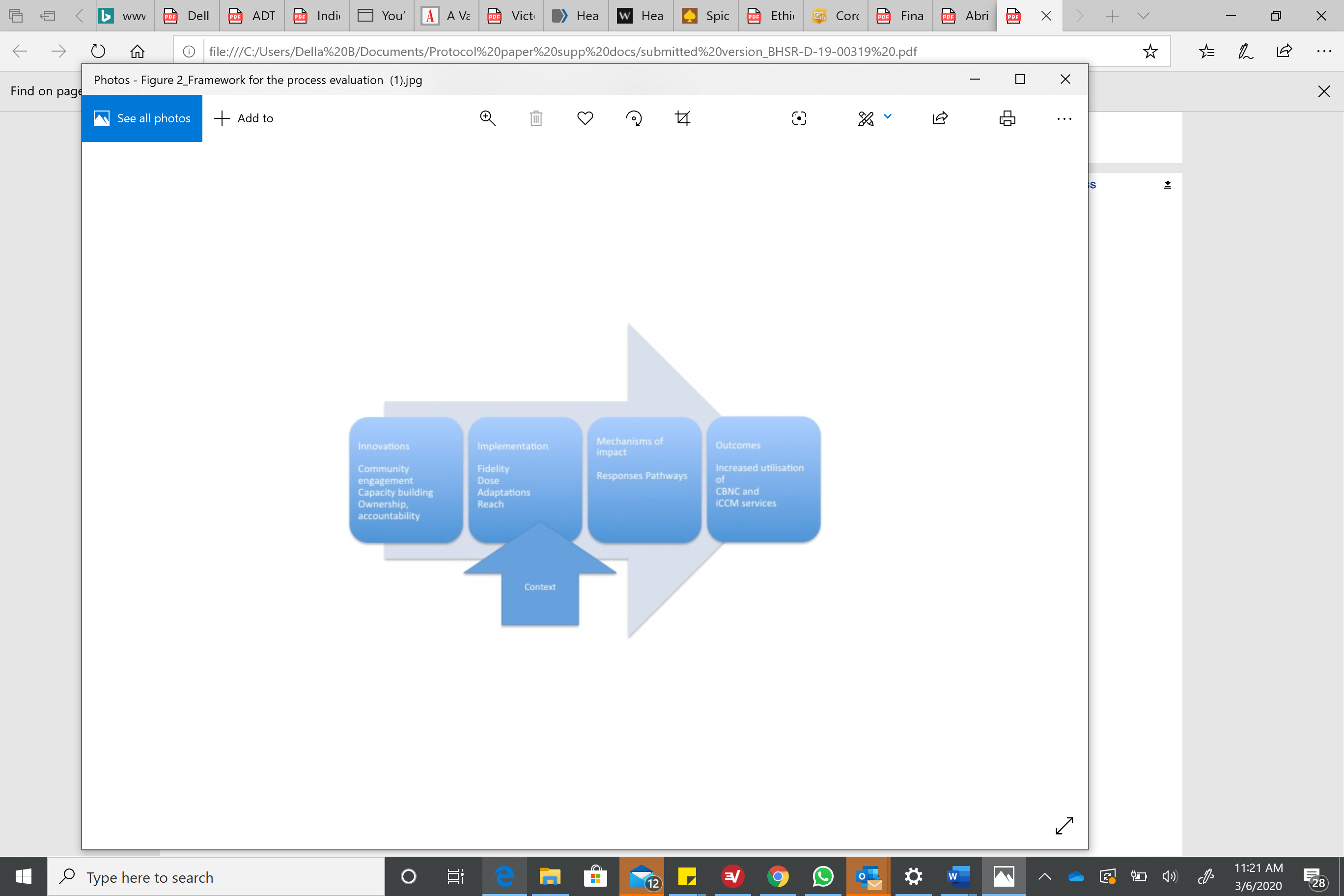


Figure 2. Framework for the process evaluation of the Optimizing the Health Extension Program intervention.

The implementing partners will prospectively collect data on the implementation of the interventions. The innovations within the three strategies will continually be harmonized across the implementing partners. Some variations between implementers and geographies is intended due to the different contexts of the districts. Such variations can be captured through the process evaluation. Implementers will also make efforts to harmonize the corresponding data sources. These databases will include information on each performed activity, including information on the innovation, the facilitator(s), the recipients, the health-system level and place, and timing of each activity. The implementing partners will also provide information on training and support to deliver the interventions and any changes made in some parts of the intervention districts or across all districts. This information will be aggregated to describe the fidelity (whether an intervention was delivered as intended), reach (how much of the intended audience was exposed to the intervention), and where feasible the dose (how much of the intervention was received by the intended audience) and adaptations (originally unintended changes made to an intervention across all or in selected parts of the study areas). In the endline survey, the participants (mothers of children below the age of five years, WDA leaders, HEWs, woreda health office representatives) will be asked about their contact with the interventions to measure reach. The baseline and endline surveys include questions on the interactions between the households and both WDA members and HEWs to measure dose (Table 2). Overall, the implementation process data will allow for analyses of whether the Optimizing the Health Extension Program innovations were implemented according to plans and associated with better awareness and acceptance of CBNC and iCCM services, provision of improved quality of services, and strengthened ownership and accountability of CBNC and iCCM services, respectively. In addition, a qualitative study will be conducted to explore views and experiences of program planners, managers and implementers, identifying what components worked well and what was not successful in the delivery of Optimizing the Health Extension Program interventions.

### Effectiveness of the Optimizing the Health Extension Program

The baseline and endline surveys in intervention and comparison areas will include modules for household, facility preparedness to provide child health services, the health worker, a quality of care assessment, the WDA leader and woreda contextual factors questionnaires. As part of the endline survey the HEWs will be interviewed, using the questionnaire-based Context Assessment for Community Health (COACH) tool (27). This validated tool measures eight aspects of the context in which the HEWs work.

Table 2. Baseline and endline survey questionnaires for the Optimizing the Health Extension Program intervention evaluation.

| Questionnaire modules | Content |
| --- | --- |
| Household  **N=6000** | - Location of household using global positioning system (GPS) coordinates - Members of household - Characteristics of the house and assets ^a^ - Women of reproductive age - Birth history - Use of maternal and perinatal health services - Knowledge of child diseases and danger signs - Care seeking and treatment for child illness - Preventive behaviour |
| Health post module  **N=200**  Halth centre module^b^ | - Location of health post and health centre using GPS coordinates - Facility-level preparedness to provide child health services - Data extracted from registers - Supportive supervision and mentorship from health centres to health posts |
| Health extension worker module **^c^** | - Knowledge on newborn and child health care - Training, supervision, mentorship - Services provided to newborns and children |
| Health centre staff module ^d^ | - Knowledge on newborn and child health care - Training, supervision - Services provided - Working conditions |
| Health provider assessment of the quality of care for a sick child module  **N=800** | - Observation and re-examination of Health Extension Workers’ assessment, classification, and treatment of sick children at health post |
| Women’s development army module  **N=200** | - Training - Knowledge - Activities in promoting maternal, newborn, and child health |
| Woreda contextual factors module  **N=52** | - Demography - Maternal, newborn and child health programs - District resources and infrastructure - Training and supervision activities, - Recent epidemics and natural disasters |
| Context Assessment for Community Health (COACH) module ^e^  **N=200** | - Available resources, - Community engagement, - Monitoring services for action, - Sources of knowledge, - Commitment to work, - Work culture, - Leadership, - Informal payment |

^a^ Asset ownership will be used to estimate relative socio-economic status, using an asset index based on principal components analysis

^b^ Some health posts are served by the same health centre hence the exact samples size can’t be determined

^c^ All the health extension workers in each health post will be interviewed. Due to the varying numbers of workers in health posts, the exact sample size can’t be determined.

^d^ We will interview one staff per sampled health centre

^e^ Conducted at endline survey only with on HEW in each health post

### Outcomes

*The primary outcome* is care seeking (at health posts, health centres, hospitals and clinics) for an illness in under-fives. *Secondary outcomes* include appropriate treatment for diarrhoeal diseases (oral rehydration therapy, zinc tablets), probable pneumonia (antibiotics), fever and malaria, and neonatal sepsis (antibiotics); improved knowledge towards childhood illnesses and treatment services among caregivers of under-5 children; improved attitudes or perceptions towards the iCCM and CBNC services at health posts; improved iCCM and CBNC program ownership by the public health sector (including inclusion of iCCM and CBNC indicators in their planning and budget allocation); and, improved availability of quality iCCM services provided by the HEWs.

*The sample size* for baseline and endline surveys is based on the requirement that these surveys should have adequate power to measure changes in a fixed number of percentage points between intervention and comparison areas from start to end of the study. For the household survey, the sample size was powered for the main outcome of care seeking for any illness in the two weeks prior to the survey. Sampling 30 households in the 100 selected enumeration areas would yield 3000 households per group. The Ethiopia Demographic Heath Survey (DHS) data has shown that the rate of children under-five to households surveyed was 0.65. Based on this assumption, a sample survey of 3000 households per group would be expected to achieve a sample size of 1747 children below the age of five years. The evaluation of the integrated management of childhood illness in Tanzania reported 50% of under-five children to have had an illness in the two weeks prior to the survey (28). We assumed a more conservative 30%. Based on the calculations of a sample size of 3000 households per group (6000 in total) with 90% completeness and a design effect of 1.3, we would have 80% power to detect differences of 10-20 percentage points across the range of child health indicators as statistically significant at the 5% level.

For the survey to assess the quality care provided by HEWs, with the assumption that each of the sampled enumeration areas will be served by one health post, the survey will include 100 intervention and 100 comparison area health posts for the quality of care assessment, where in each health post, the HEWs’ assessment of four sick children mobilized to come to the health post will be observed, followed by a re-examination of the child by a health officer. This will yield a sample size of 400 children in each group. A total of 800 children, with a design effect of 1.4, will have 80% power to detect a minimum of 15 percentage point changes in the correct classification of iCCM illnesses between intervention and comparison area HEWs at baseline and endline as statistically significant at the 5% level.

*The questionnaire tools for baseline and endline surveys* were adapted from the team’s own, and others’ previous work on the Integrated Management of Childhood Illness (28), iCCM (29), and CBNC (30). The questionnaire instrument includes modules regarding household, health posts and health centres, HEWs, health centre staff, WDA members, an observation and re-examination of the HEW assessing sick children, and district contextual factors (Table 2).

*The baseline and endline surveys* will be conducted in intervention and comparison areas to assess the situation before the intervention. A two-stage stratified cluster sampling will be applied in these two surveys using lists of enumeration areas of the 52 intervention and comparison districts from the latest (2007) Ethiopian census as the sampling frame. In the first stage, a list of all enumeration areas of the study districts will be based on the 2007 Ethiopian Housing and Population Census. Two hundred enumeration areas will be selected from 52 districts with probability proportional to size. Each enumeration area will form one cluster, and these clusters will constitute the primary sampling unit. In the second stage, a systematic random sampling technique will be applied to select 30 households in each cluster. All women aged 13 to 49 years who live in the selected households will be included, in order to identify women who had a live birth in the 12 months prior to the survey to assess care seeking in the neonatal period. Furthermore, children under the age of 5 years will be included to assess care seeking for any illness in the two weeks prior to the survey. For every cluster, the WDA leader serving the cluster will be interviewed. The health post and the HEWs serving the selected cluster, the health post’s referral health centre, and staff, and the district health office providing support to the selected facility will be approached with survey modules. All study participants will be sampled without replacement. Up to three visits will be made to each participant to maximize their inclusion into the study.

To evaluate the quality of assessment and care provided to sick children at the health posts, an observation of a sick child consultation with an HEW and a re-examination by a child health officer will be performed. Given that very few sick children are brought to the health post each day, data collectors will mobiliz the community to bring sick children on the day of the survey to ensure the required sample size is met.

Data collection teams and supervisors will be trained over the course of ten days. They will not be provided information on whether a district is an intervention or comparison area. Data will be collected on tablets and encrypted data will be regularly sent from the field to the Ethiopian Public Health Institute’s central server. The data manager will then decrypt the data and rigorous quality checks will be conducted with feedback to the field teams. Data cleaning will involve checking for errors, completeness and consistency. The data manager will also ensure that all standards for data security, curation and access were met.

*The effectiveness assessment* will be based on a plausibility design (16), analysing difference-in-differences of the primary and secondary outcomes (31). Data on primary and secondary outcomes will be analysed from baseline and endline household, health post, and health centre surveys in intervention and comparison districts, with adjustment for the cluster sampling and relevant confounding factors. The assessment will use blinded analysis. The code identifying the intervention and comparison areas will be revealed after the analysis and interpretations are completed.

Ten Ph.D. students from four Ethiopian universities, including candidates from the Regional Health Bureaus in the study provinces, and from the Ethiopian Public Health Institute, are engaged in the evaluation. PhD students will be involved in the conduct of the surveys, participating in the training of data collectors and serving as regional survey coordinators. They have chosen topics for in-depth sub-studies linked to the evaluation, including equity in the utilisation of services; spatial analyses of care utilisation; quality of care provided by the HEWs; the role of WDA leaders in promoting the use of services; newborn care practices; the referral of sick children; and focused studies of care utilisation for diseases of the newborn and diarrhoeal diseases. Most of the students plan to use baseline and endline surveys for quantitative data and to perform qualitative studies within their chosen topics.

Findings from the effectiveness study, process evaluation and PhD research will be published as scientific articles and reports. No professional writers will be used. Publications arising from this evaluation will follow the recommendations from the International Committee of Medical Journal Editors (32).

The interventions started in the 4^th^ quarter of 2016 and will run up to the end of 2018. Baseline survey will be conducted in the 4^th^ quarter of 2016 and endline survey will start immediately thereafter the end of the program implementation. Data for process evaluation collection and analysis will take place until September 2019. Data analysis for the effectiveness study will be performed from mid of 2019 onwards.

### Ethics approval and consent to participate

Data collectors will obtain written informed consent from all interviewees. For women between the ages of 13-17 years to be included in the household survey, assent will be obtained from a parent or guardian. The Ethiopian Public Health Institute Ethical Review Board and the London School of Hygiene & Tropical Medicine Ethical Review Board approved the evaluation (LSHTM Ethics Ref 16117 and EPHI Ethics Ref 613/52). Any deviations to the approved protocol will be reported to the Ethical Review Boards of both countries.

## Conclusion

This protocol describes the evaluation of a complex intervention in four regions of Ethiopia that aims at increasing utilisation of primary care services for mothers and children. The intervention is based on an analysis of barriers to the utilisation of services and includes innovations to engage communities, train and support primary care workers, and promote ownership and accountability of CBNC and iCCM programs. Representatives of the health system, non-governmental organisations, and universities work together to learn, evaluate, and strengthen university capacity for health systems and implementation research, which will enhance sustainable work relationship between universities and Regional Health Bureaux. The process and outcome evaluations will inform the possible scale-up of efforts to increase primary health care utilisation for sick children in Ethiopia and similar settings.

References

1. Ruducha J, Mann C, Singh NS, Gemebo TD, Tessema NS, Baschieri A, et al. How Ethiopia achieved Millennium Development Goal 4 through multisectoral interventions: a Countdown to 2015 case study. The Lancet Global Health. 2017 Nov;5(11):e1142–51.

2. Corsi DJ, Neuman M, Finlay JE, Subramanian S. Demographic and health surveys: a profile. Int J Epidemiol. 2012 Dec;41(6):1602–13.

3. Deribew A, Tessema GA, Deribe K, Melaku YA, Lakew Y, Amare AT, et al. Trends, causes, and risk factors of mortality among children under 5 in Ethiopia, 1990-2013: findings from the Global Burden of Disease Study 2013. Popul Health Metr. 2016;14(1):42.

4. Yitayal M, Berhane Y, Worku A, Kebede Y. Health extension program factors, frequency of household visits and being model households, improved utilization of basic health services in Ethiopia. BMC Health Serv Res. 2014 Apr 5;14(1):156.

5. Miller NP, Amouzou A, Tafesse M, Hazel E, Legesse H, Degefie T, et al. Integrated community case management of childhood illness in Ethiopia: implementation strength and quality of care. Am J Trop Med Hyg. 2014 Aug;91(2):424–34.

6. Mathewos B, Owen H, Sitrin D, Cousens S, Degefie T, Wall S, et al. Community-Based Interventions for Newborns in Ethiopia (COMBINE): Cost-effectiveness analysis. Health Policy Plan. 2017 Oct 1;32(suppl_1):i21–i32.

7. Degefie Hailegebriel T, Mulligan B, Cousens S, Mathewos B, Wall S, Bekele A, et al. Effect on Neonatal Mortality of Newborn Infection Management at Health Posts When Referral Is Not Possible: A Cluster-Randomized Trial in Rural Ethiopia. Glob Health Sci Pract. 2017 Jun 27;5(2):202–16.

8. Girmaye M, Berhan Y. Skilled Antenatal Care Service Utilization and Its Association with the Characteristics of Women's Health Development Team in Yeky District, South-West Ethiopia: A Multilevel Analysis. Ethiop J Health Sci. 2016 Jul;26(4):369–80.

9. Jackson R, Tesfay FH, Gebrehiwot TG, Godefay H. Factors that hinder or enable maternal health strategies to reduce delays in rural and pastoralist areas in Ethiopia. Trop Med Int Health. 2017 Feb;22(2):148–60.

10. Mezmur M, Navaneetham K, Letamo G, Bariagaber H. Socioeconomic inequalities in the uptake of maternal healthcare services in Ethiopia. BMC Health Serv Res. 2017 May 22;17(1):367.

11. Bobo FT, Yesuf EA, Woldie M. Inequities in utilization of reproductive and maternal health services in Ethiopia. Int J Equity Health. 2017 Jun 19;16(1):105.

12. Tesfaye B, Mathewos T, Kebede M. Skilled delivery inequality in Ethiopia: to what extent are the poorest and uneducated mothers benefiting? Int J Equity Health. 2017 May 16;16(1):82.

13. Amouzou A, Hazel E, Shaw B, Miller NP, Tafesse M, Mekonnen Y, et al. Effects of the integrated Community Case Management of Childhood Illness Strategy on Child Mortality in Ethiopia: A Cluster Randomized Trial. Am J Trop Med Hyg. 2016 Mar;94(3):596–604.

14. Ayalneh AA, Fetene DM, Lee TJ. Inequalities in health care utilization for common childhood illnesses in Ethiopia: evidence from the 2011 Ethiopian Demographic and Health Survey. Int J Equity Health. 2017 Apr 21;16(1):67.

15. Skaftun EK, Ali M, Norheim OF. Understanding inequalities in child health in Ethiopia: health achievements are improving in the period 2000-2011. PLoS ONE. 2014;9(8):e106460.

16. Habicht JP, Victora CG, Vaughan JP. Evaluation designs for adequacy, plausibility and probability of public health programme performance and impact. Int J Epidemiol. 1999 Feb;28(1):10–8.

17. Moore GF, Audrey S, Barker M, Bond L, Bonell C, Hardeman W, et al. Process evaluation of complex interventions: Medical Research Council guidance. BMJ. 2015 Mar 19;350(mar19 6):h1258–8.

18. Nigatu SG, Worku AG, Dadi AF. Level of mother's knowledge about neonatal danger signs and associated factors in North West of Ethiopia: a community based study. BMC Res Notes. 2015 Jul 19;8(1):309.

19. Degefie T, Amare Y, Mulligan B. Local understandings of care during delivery and postnatal period to inform home based package of newborn care interventions in rural Ethiopia: a qualitative study. BMC Int Health Hum Rights. 2014 May 19;14(1):17.

20. Tefera W, Tesfaye H, Bekele A, Kayessa E, Waltensperger KZ, Marsh DR. Factors influencing the low utilization of curative child health services in Shebedino District, Sidama Zone, Ethiopia. Ethiop Med J. 2014 Oct;52 Suppl 3:109–17.

21. Shaw B, Amouzou A, Miller NP, Tafesse M, Bryce J, Surkan PJ. Access to integrated community case management of childhood illnesses services in rural Ethiopia: a qualitative study of the perspectives and experiences of caregivers. Health Policy Plan. 2016 Jun;31(5):656–66.

22. King R, Jackson R, Dietsch E, Hailemariam A. Barriers and facilitators to accessing skilled birth attendants in Afar region, Ethiopia. Midwifery. 2015 May;31(5):540–6.

23. Medhanyie A, Spigt M, Kifle Y, Schaay N, Sanders D, Blanco R, et al. The role of health extension workers in improving utilization of maternal health services in rural areas in Ethiopia: a cross sectional study. BMC Health Serv Res. 2012 Oct 8;12(1):352.

24. Tilahun H, Fekadu B, Abdisa H, Canavan M, Linnander E, Bradley EH, et al. Ethiopia's health extension workers use of work time on duty: time and motion study. Health Policy Plan. 2017 Apr 1;32(3):320–8.

25. Gobezayehu AG, Mohammed H, Dynes MM, Desta BF, Barry D, Aklilu Y, et al. Knowledge and skills retention among frontline health workers: community maternal and newborn health training in rural Ethiopia. J Midwifery Womens Health. 2014 Jan;59 Suppl 1(s1):S21–31.

26. Berhe AK, Tinsae F, Gebreegziabher G. Knowledge and practice of immediate newborn care among health care providers in eastern zone public health facilities, Tigray, Ethiopia, 2016. BMC Pediatr. 2017 Jul 11;17(1):157.

27. Bergström A, Skeen S, Duc DM, Blandon EZ, Estabrooks C, Gustavsson P, et al. Health system context and implementation of evidence-based practices—development and validation of the Context Assessment for Community Health (COACH) tool for low- and middle-income settings. Implement Sci. 2015 Aug 15;10(1):120.

28. Armstrong Schellenberg JRM, Adam T, Mshinda H, Masanja H, Kabadi G, Mukasa O, et al. Effectiveness and cost of facility-based Integrated Management of Childhood Illness (IMCI) in Tanzania. Lancet. 2004 Nov;364(9445):1583–94.

29. Marsh DR, Nefdt R, Hazel E. Introduction to a special supplement: delivering integrated community case management to treat childhood illness at scale in Ethiopia. Ethiop Med J. 2014 Oct;52 Suppl 3:1–6.

30. Berhanu, D. Community Based Newborn Care: Baseline report summary, Ethiopia October 2014. London: IDEAS, London School of Hygiene & Tropical Medicine. https://ideas.lshtm.ac.uk/wcpcontent/uploads/2017/08/CBNCBaselineReport_18nov15_WEB.pdf

31. Dimick JB, Ryan AM. Methods for evaluating changes in health care policy: the difference-in-differences approach. JAMA. 2014 Dec 10;312(22):2401–2.

32. Uniform requirements for manuscripts submitted to biomedical journals. International Committee of Medical Journal Editors. Vol. 277, JAMA. 1997. pp. 927–34.
