# Supplementary material for: Association between a complex community intervention and quality of health extension workers’ performance to correctly classify common childhood illnesses in four regions of Ethiopia
Source: PLoS One. 2021 Mar 12;16(3):e0247474. doi: 10.1371/journal.pone.0247474 (PMC7954333; doi:10.1371/journal.pone.0247474)
Supplement: Attachment — Submitted filename: 3. Response to reviewers_.docx [file pone.0247474.s004.docx]

Jun 11, 2020

**PLOS ONE**

Dear Editor,

Thank you for giving us the opportunity to submit a revised manuscript again, PONE-D-20-05286, “Association between a complex community intervention and quality of health extension workers’ performance to correctly classify common childhood illnesses in four regions of Ethiopia”.

We appreciate the time and effort that you and the reviewers have devoted to provide valuable feedback on the manuscript. We are grateful to the reviewers for their insightful comments on the paper. We have incorporated the changes based on all the suggestions provided by the reviewers.

The map used to indicate the study area was produced by the authors using the open access reliable web site “GADM” available at <https://gadm.org/download_country_v3.html>. Now, we have written the source data used for production as a footnote. We have indicated the amendment using track changes in the manuscript.

Any reasonable data request will get access to data after contacting Della Berhanu, [della.berhanu@lshtm.ac.uk](mailto:della.berhanu@lshtm.ac.uk). The data policy of the Ethiopian Public Health Institute does not allow us to put these data to immediate access at a public repository.

Following is a point-by-point response to the reviewers’ comments and concerns and we hereby submit the revised work for your consideration.

Thank you for considering our work.

Theodros Getachew

Corresponding author on behalf of the authors

PONE-D-20-05286
Association between a complex community intervention and quality of health extension workers’ performance to correctly classify common childhood illnesses in four regions of Ethiopia
PLOS ONE

3. Please include a caption for figure 1.

- Thank you. We have added the caption. Page 5 line 105.

4. We note that Figure 1 in your submission contain [map/satellite] images which may be copyrighted. All PLOS content is published under the Creative Commons Attribution License (CC BY 4.0), which means that the manuscript, images, and Supporting Information files will be freely available online, and any third party is permitted to access, download, copy, distribute, and use these materials in any way, even commercially, with proper attribution. For these reasons, we cannot publish previously copyrighted maps or satellite images created using proprietary data, such as Google software (Google Maps, Street View, and Earth). For more information, see our copyright guidelines: http://journals.plos.org/plosone/s/licenses-and-copyright.

We require you to either (1) present written permission from the copyright holder to publish these figures specifically under the CC BY 4.0 license, or (2) remove the figures from your submission:

- Thank you. However, the map was produced by the authors and the source of the data files was cited as a footnote to the map.

Additional Editor Comments (if provided):

The paper is well written and just requires minor revisions as recommended by Reviewer1 which I agree with. The Conclusion should include some form f a policy and/or program recommendation or perhaps a more randomized clinical trial to re-validate the negative findings or perhaps a RCT may show different results for the intervention evaluated.

- Thank you. We have added our reflections in the conclusion. Page 15 line 303.

Can the authors explain the absence of data on fever/malaria at endline in the comparison group in Fig 3?

- Thank you. It was an unintentional mistake. We have now added this in Figure 3.

Reviewers' comments:

Reviewer's Responses to Questions

**Comments to the Author**

5. Review Comments to the Author

Reviewer #1: This paper is of high quality and well presented. My reservation is how useful is it to the Ethiopian health planners? While the literature quoted is good, most comments are at an overview level. Are there any specific recommendations that could be presented as to the way forward from such papers that describe successful interventions. Such as White EE, Downey J Sathananthan et al (your Ref 27) , Feyissa GT, Balabanova, Woldie M et al (your Ref 9) The latter papers (28 onwards) indicate the authors have a lot of experience in Ethiopia that suggests that specific recommendations may be useful. Also line 410, Leatherman S reference is incomplete -Journal lacking. Thanks for your work.

- Thank you. We have added our reflections based on the findings in the conclusion. Page 15 line 303.
- The Leatherman S reference is now corrected
