# Supplementary material for: Association between a complex community intervention and quality of health extension workers’ performance to correctly classify common childhood illnesses in four regions of Ethiopia
Source: PLoS One. 2021 Mar 12;16(3):e0247474. doi: 10.1371/journal.pone.0247474 (PMC7954333; doi:10.1371/journal.pone.0247474)
Supplement: Attachment — Submitted filename: PONE-D-20-05286R2 r2r.docx [file pone.0247474.s005.docx]

**Comments to the Author**

1. If the authors have adequately addressed your comments raised in a previous round of

review and you feel that this manuscript is now acceptable for publication, you may indicate

that here to bypass the “Comments to the Author” section, enter your conflict of interest

statement in the “Confidential to Editor” section, and submit your "Accept"

recommendation.

Reviewer #2: (No Response)

2. Is the manuscript technically sound, and do the data support the conclusions?

The manuscript must describe a technically sound piece of scientific research with data that

supports the conclusions. Experiments must have been conducted rigorously, with

appropriate controls, replication, and sample sizes. The conclusions must be drawn

appropriately based on the data presented.

Reviewer #2: No

• Response. We are thankful to the reviewer for the comments below related to this

statement and have added details in the description of the methods used in the

analyses. We have revised the management of potential confounding in the analyses

(see below). With these revisions, we consider the analyses as technically sound

with data that support the conclusions.

3. Has the statistical analysis been performed appropriately and rigorously?

Reviewer #2: No

• Response. In the revised version of the manuscript we have revised the management

of potential confounders. We believe that sufficient details now are provided in the

methods section. We consider the statistical analyses appropriate and rigorously

done.

4. Have the authors made all data underlying the findings in their manuscript fully available?

The PLOS Data policy requires authors to make all data underlying the findings described in

their manuscript fully available without restriction, with rare exception (please refer to the

Data Availability Statement in the manuscript PDF file). The data should be provided as part

of the manuscript or its supporting information, or deposited to a public repository. For

example, in addition to summary statistics, the data points behind means, medians and

variance measures should be available. If there are restrictions on publicly sharing data—

e.g. participant privacy or use of data from a third party—those must be specified.

Reviewer #2: Yes

5. Is the manuscript presented in an intelligible fashion and written in standard English?

PLOS ONE does not copyedit accepted manuscripts, so the language in submitted articles

must be clear, correct, and unambiguous. Any typographical or grammatical errors should

be corrected at revision, so please note any specific errors here.

Reviewer #2: Yes

6. Review Comments to the Author

Please use the space provided to explain your answers to the questions above. You may also

include additional comments for the author, including concerns about dual publication,

research ethics, or publication ethics. (Please upload your review as an attachment if it

exceeds 20,000 characters)

Reviewer #2: This manuscript is really lacking in details regarding the statistical methods.

Propensity score matching is proposed in the abstract and then barely touched upon in the

methods. It's unclear how this improves the causal inference between the intervention

groups in the article and results in a very confusing and problematic methods section.

• Response We are thankful to the reviewer for these comments. In the revised

version of the manuscript we have revised the data analysis done to accommodate

the clustering effect without propensity score matching. The estimates were only

marginally influenced. We now consider the statistical analyses appropriate and

rigorously done and hope these modifications are satisfactory.

1. (lines 107-114) I would probably combine this section with the Sampling subsection.

• Response. Thank you. This study is a non-randomized trial. The journal requires that

a TREND checklist is used in preparing the manuscript and according to the checklist,

study participants should appear as the first subheading. This section should address

eligibility criteria for participants, including criteria at different levels in

recruitment/sampling plan, method of recruitment including the sampling method,

and recruitment setting. Therefore, we prefer to keep it as a separate section.

2. (line 109) More details on stratified cluster sampling are needed. For instance, how were

strata defined? How many enumeration areas were sampled per stratum? How were EAs

and health posts selected?

• Response. Thank you for the comments. We have added a detailed description on

the stratifying sampling strategy in line number 112-114.

3. (lines 178-180) What type of regression model?

• Response. Thank you. We include a description of the regression in line 186-192.

4. (lines 180-181) Sadly, there is very little information here on the propensity score

methods. This is mentioned in the abstract and is implied to be a key part of your

methodology, yet there is one sentence in the methods. This absolutely must be described

in greater detail to satisfy #3 of the PLOS criteria for publication

(https://journals.plos.org/plosone/s/criteria-for-publication). For instance, this article

(https://www.sciencedirect.com/science/article/pii/S0002914903012839) is one I have used

as guide, except that having p-values in table 1 is now not looked upon favorably. If you are

using propensity scores, you are implying that you have covariate imbalances in between

your groups. Thus, you need to demonstrate how the propensity scores have improved the

balance between groups. You also need to carefully lay out your propensity score

implementation. This includes methodological citations for your methods, i.e., the

estimation of the propensity scores and, especially, the matching as there are many

different ways to do this. You must articulate what was used to estimate the propensity

scores.

• Response. Thank you. As mentioned above, we have revised our analysis method

and in this version of the manuscript propensity score method is not included. The

methods used to manage potential confounding and adjust for cluster sampling are

described in line 186-192.

• In this paper, we used the features of the user-written command diff in STATA,

which estimates difference-in-differences (DID) treatment effects. diff simplifies the

DID analysis by allowing the conventional DID setting to be combined with other

nonexperimental evaluation methods.

5. (line 183) Each of the parameters in this equation need to be defined. I'm guessing Y is

described in lines 184-187, but this is never stated.

• Thank you. We have now defined the parameters, line 196-204.

6. (line 183) In theory, the propensity score matching should mean that no other covariates

are needed in the regression model. It's unclear why covariates are needed in this final

model.

• Response. We have, as mentioned, revised our analysis to a conventional DID. In the

analysis our dependent variable is correct disease classification of childhood

illnesses, which mainly could be confounded by the age of the child.

7. Nowhere in the methods is it mentioned that clustering from the enumeration areas. This

must be done since there may be differences between individuals sampled from one area

compared to another.

• Response. Thank you. We have revised the analysis to multilevel mixed regression to

consider the potential clustering effect at health posts and at health extension

worker level, see line 264-267.

8. Please indicate the level of significance for these analyses.

• Thank you. We have indicated the significance level in line 192.

9. (Table 1) I don't understand why there are CIs on these numbers. They are facts about the

sample and not estimated.

• Response. The purpose of including CIs in table 1 is that we provide a range of values

with a known probability of capturing the population parameter from our sample

parameter. In this case, we claim to have 95% confidence that it will include the true

population parameter for the selected study areas.

10. (line 253-255) This is speculative. If you want to make this statement, why not try to

support it with numbers collected from a survey that was designed to be representative?

• Response. Yes, we agree it is a speculative. This paragraph discussed the external

validity of the study to indicate that the initial assumption of sampling was not to

represent the regions. In this case, we are not able to support this with a number.

11. (lines 255-256) How do you define similar? Superiority significance testing or 95% CIs are

not acceptable here. I could conclude they are different; for instance, I could conclude that

39% of the intervention sample being from 2-11 month olds is different from 26% in the

comparison sample.

• Response. Thank you. We have rephrased the sentence as “The selection of

intervention and comparison districts provided similar study population

characteristics at baseline, except for the age category.” Line 283-284.

• This was the rationale for including age as a covariate in the analyses.

12. (Figure 3 and 4) Please include confidence intervals on these figures.

• Response. Thank you. We have now added CIs in the figures.
